# Peer-Mediated Multimodal Intervention Program for the Treatment of Children with ADHD in India: One-Year Followup

**DOI:** 10.5402/2012/419168

**Published:** 2012-12-20

**Authors:** Sagar Mehta, Devesh Shah, Kushal Shah, Sanjiv Mehta, Neelam Mehta, Vivek Mehta, Vijay Mehta, Vaishali Mehta, Smita Motiwala, Naina Mehta, Devendra Mehta

**Affiliations:** ^1^Trinity College of Arts and Sciences, Duke University, Durham, NC 27708, USA; ^2^Barnet General and Chase Farm Hospital, Wellhouse Lane, Barnet, Hertfordshire EN5 3DJ, UK; ^3^Imperial College London, South Kensington Campus, London SW7 2AZ, UK; ^4^Perelman School of Medicine, University of Pennsylvania, Philadelphia, PA 19104, USA; ^5^University of Miami, Coral Gables, FL 33124, USA; ^6^New York University, New York, NY 10012, USA; ^7^Lake Erie College of Osteopathic Medicine, Bradenton, FL 34243, USA; ^8^Winter Park High School, Winter Park, FL 32792, USA; ^9^Harsh Vardhan Memorial Charitable Trust, Safdarjung Enclave, New Delhi 110029, India; ^10^Children's Medical Services, 7000 Lake Ellenor Dr, Orlando, FL 32809, USA; ^11^Arnold Palmer Hospital for Children, 92 West Miller Street, Orlando, FL 32806, USA; ^12^Florida State University Regional Medical School Campus, 250 East Colonial Drive, Suite 200, Orlando, FL 32801, USA

## Abstract

The objective was to assess the efficacy of a one-year, peer-mediated interventional program consisting of yoga, meditation and play therapy maintained by student volunteers in a school in India. The population consisted of 69 students between the ages of 6 and 11 years, previously identified as having attention deficit hyperactivity disorder (ADHD). A program, known as Climb-Up, was initially embedded in the school twice weekly. Local high school student volunteers were then trained to continue to implement the program weekly over the period of one year. Improvements in ADHD symptoms and academic performance were assessed using Vanderbilt questionnaires completed by both parents and teachers. The performance impairment scores for ADHD students assessed by teachers improved by 6 weeks and were sustained through 12 months in 46 (85%) of the enrolled students. The improvements in their Vanderbilt scores assessed by parents were also seen in 92% (*P* < 0.0001, Wilcoxon). The Climb-Up program resulted in remarkable improvements in the students' school performances that were sustained throughout the year. These results show promise for a cost-effective program that could easily be implemented in any school.

## 1. Introduction

 Attention deficit hyperactivity disorder (ADHD) is one of the most common disorders of childhood, affecting 5.3% to 20% of the children worldwide [1, 2]. US studies have shown a prevalence of 8.7% in 8–15-year olds [3]. Other studies in India based on hospital or outpatient clinic populations, with referral bias, suggest prevalence of 5.2% to 29.5% [4–6]. 

 The condition leads to poor academic performance at school and problems with behavior at home and school. Children with this disorder often have comorbid conditions such as oppositional defiant disorder and learning disabilities, which all adversely impact the family and community. As they reach adolescence, these children are also at greater risk of drug and alcohol abuse and other issues such as increased rate of motor vehicle accidents, adding a substantial cost burden to the society. These children also suffer from problems with family and peer relationships that continue into adulthood and prevent the individual from achieving their maximum potential [7]. 

 Studies in small settings have shown that peer tutoring does help children with ADHD [8, 9]. Peers can offer role models, have closer associations with the children throughout school, help build self-esteem, and provide a more suitable environment for the needs of the ADHD students [10]. 

 Yoga with meditation modified for children was chosen as the physical exercise component. Yoga also needs limited space, no equipment, is easy to learn, and also culturally well accepted. There is strong belief but limited evidence to show that yoga and meditation help focus and attention. Pilot studies using this as family based therapy for ADHD in 8 boys have been reported to show promise [11]. Additional theoretical basis may be the increase in dopamine release in the CNS from yoga [12]. In ADHD, reduced dopamine levels are seen in the CNS, and strategies to regulate these levels are suggested as possible therapies [13]. 

 Treatment with behavioral therapy with or without medications leads the children to do well at school and college and become productive adults. Multimodal behavioral programs integrating play therapy, exercise, and reward systems using psychologists have been shown to help the majority of children with ADHD [14]. We established a pilot project incorporating yoga and meditation in a multimodal behavioral program called Climb-Up, and reported our 6 week intervention findings recently [15]. We noted similar global improvement irrespective of the type of ADHD. However, as others have found, once intensive therapy is discontinued, relapses occur [16]. The solution we devised however was potentially a self-sustaining, low cost program not dependent on scarce professionals such as psychologists or even teachers. One strength was that this could also be readily embedded into the school curriculum to ensure compliance. We now report the 6-month and one-year-follow up data to assess whether these early benefits were sustained without direct external resources.

## 2. Method

### 2.1. Population

The study population studied consisted of children in 2nd to 5th grades (ages 6 yrs to 11 yrs) identified as having ADHD, attending a school in Najibabad, a small town 400 kilometers north of Delhi. IRB approval for the project was granted by the school board of MDKV and registered under clinicaltrials.gov (NCT01012778). All 910 children ageing 6 to 11 were screened for ADHD using the Initial Teacher Vanderbilt Assessment. As reported previously, 80 children were identified, using Vanderbilt questionnaires as the assessment tool and a specific diagnosis made by a neurodevelopment pediatrician using DSM IV criteria [15] (see Appendix). Parents of 76 of these children signed informed consent forms and agreed to have their child enroll, 70 had completed initial teacher evaluations, and 69 stayed in the program consistently for 12 months. Of the seven lost to followup, five moved to other schools, and two came intermittently and were excluded from the data analysis. Of these, 14 had coexisting oppositional defiant disorder (ODD).

### 2.2. Population Characteristics

 The town's population is approximately 155,000 with about 55% of the children attending the public boys or girls schools. The other 45% children attended a Catholic or private school in the town, boarding schools elsewhere, or did not attend school and were not part of our survey. The population consisted of just over 50% Muslim and 48% Hindu, in keeping with the state average. Also about 90% of the population lived in the urban area of the town with the other 10% living in the rural area. There are three pediatricians and several general practitioners in the town [17, 18]. 

 This program was implemented in Najibabad over a 6-week period. The School Board formally approved the study. The interventional program was devised as part of a program called Climb-Up. The team originally consisted of a neurodevelopmental pediatrician to evaluate the children for ADHD and comorbidities, a pediatrician to evaluate general health, a coordinator, two teachers as translators, and seven external student volunteers to help manage the flow, collect and enter data, and provide the multimodal peer-mediated interventions. These students then taught local high school volunteers the yoga poses and simple meditation techniques using breathing suitable for children. These were selected from Prekshya Dhyana, a Jain approach to yoga and meditation that emphasizes deep breathing techniques [19]. Games that are typically used to increase attention span, while improving their memory, concentration, and social skills, were translated into Hindi with culturally appropriate modifications made as necessary. 

 The children with ADHD were divided into age- and sex based groups of 8 to 10 each. Twice weekly morning sessions were established consisting of 25 minutes of yoga and meditation, followed by 30- minute play therapy, and a 5-minute discussion/feedback from the children. Each group had 2 external student volunteers delivering the program initially. Following 6 weeks of behavioral therapy, 69 children remained in the program. Out of the remainder, an incomplete response was seen in 14 of the 69 children as identified by the developmental pediatrician at 6 weeks, based on DSM IV criteria (see Appendix), who were started on Methylphenidate in addition to the peer-mediated program. These children had significant ongoing symptoms of inattention and hyperactivity in the class that was disruptive, and 57% (8/14) of them had ODD. These children were excluded from the analysis, as medications would be a confounder. The remaining 55 were followed for 12 months and were not different from the original 70 in terms of type of ADHD (67.1% combined, 21.4% inattentive, and 11.4% hyperactive/impulsive). Assessment for dyslexia was attempted, and distribution between medicated (3/14) and not medicated (7/55) was similar. However, poor literacy and a lack of reading material at home during preschool made this difficult to define and it was therefore omitted from analysis. Attendance and level of involvement in the program were tracked and provided graphically to give each child feedback. In addition, tangible rewards were given each session to spur ongoing effort. During this time 13 local high school 11th graders were recruited. These high school volunteers were trained during weeks 3 and 4, and they then ran the program for the last 2 weeks with observation and feedback from the Climb-Up team. A yoga and meditation proficiency test was administered toward the end confirming that all children had reasonable skills [15]. The volunteers continued the program twice weekly per group for 2 more weeks and then once weekly throughout the year, except during school breaks, examination week, and the first week of a new term. During the winter months, the class time switched to the last class before lunch. The volunteer class frequency was logged and signed off by the school principal. This record was sent monthly to us, and upon receipt, positive feedback was provided to the volunteers in the form of community service hours earned. In December 2007, a one-week session to obtain their feedback and change games used as play therapy was instituted by a trained member of the intensive intervention group. In July 2008, an award ceremony was established with the cooperation of the town Mayor, signed volunteer certificates were distributed, and a token award of school supplies or cloth was given. The children were originally screened for ADHD using the Initial Teacher Vanderbilt Assessment [7] in May 2007. Performance Impairment Scores are only obtained by the teachers on the Vanderbilt questionnaire and are based on 8 areas: reading, mathematics, written expression, relationship with peers, following directions, disrupting class, assignment completion, and organizational skills. Each category is scored from a 1 to 5 with a 4 or 5 indicating impairment and abnormal scores [7]. The number of areas of impairment is then tallied (maximum 8) to give the Performance impairment score. Initial and 6-week followup teacher assessments were completed by a total of 8 teachers that led classes for this age group of children. Likewise initial and followup parent assessments were completed at the onset and then subsequently completed after 6 weeks of the program (July 2007). One further parent followup was completed at 6 months (December 2007) and a further teacher followup by the same teachers at the beginning of the next school year (July 2008) [15]. A translator administered the questionnaires with copies translated into Hindi by trained teachers. The parents with limited literacy were assisted by teachers of other classes to help complete the questionnaire. Though not specifically validated in India in a population with high levels of illiteracy, this score system has been used in other developing countries as well as by others in India [20, 21]. The scores are generated for hyperactivity and inattentiveness, and these are combined to give the Vanderbilt raw score, as reported elsewhere [15]. Comorbidities including oppositional defiant disorder (ODD) and dyslexia were also evaluated. Data were collected using Excel and Epi Info Database. Data entry was performed from paper records and checked for accuracy at time of initial analysis. Data sets for children without medication are presented. These data were analyzed for all the enrolled children as an aggregate group, and specific age groups were not separately analyzed due to the resulting smaller sample size. Analysis was performed using descriptive statistics, and in view of the skewed nonnormal distribution and wide standard deviations, Wilcoxon nonparametric test for multiple comparisons for each time point was used. Significance was determined as *P* value <0.05.

## 3. Results

 There were no significant differences in the demographic features, nutritional statuses, socioeconomic patterns, or the religions between those enrolled children and their families and those children and families not enrolled. 

**Figure 1:**
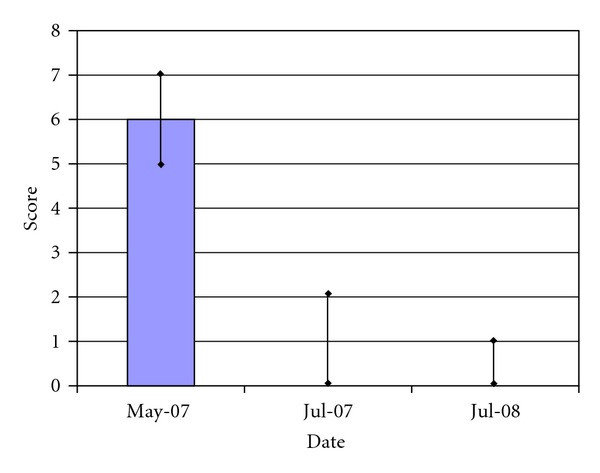
Performance impairment scores for children with ADHD in Climb-Up program (*N* = 55). The figure shows the teachers' performance impairment median scores for children in the Climb-Up program at baseline (May 2007), 6-week (July 2007), and 1-year (July 2008) followup. The bar indicates the 25% to 75% quartiles for each data. The difference between the baseline value (May 2007) and both follow-up assessments is significant at *P* < 0.001.

 Performance impairment is assessed by the teachers only. The follow-up performance impairment scores were completed at 6 weeks in July 2007 and one year July 2008. 55 had completed all three assessments (Figure 1). A total of 8 teachers completed these assessments for each of the children enrolled in their classes. Intraobservational analysis could not be conducted as there was only a single teacher per class, and teachers were unable to assess children outside of their class; however we did not see any differences in percent abnormal assessments per teacher. For the children without medication in the program, the performance impairment score decreased remarkably from median 6, range 3–8 at baseline in May 2007 to median 0, range 0–7 in July 2007 (*P* < 0.001 Wilcoxon signed rank test). This improvement from baseline was sustained with the median score of 0, range 0–8 in July 2008 (*P* < 0.001 Wilcoxon signed rank test, from baseline). 

**Figure 2:**
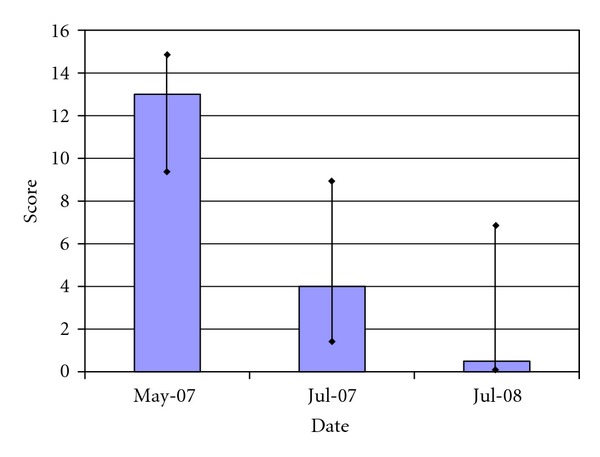
Teacher raw Vanderbilt scores for children in Climb-Up program (*N* = 55). The figure shows the teachers' raw Vanderbilt median scores for children with ADHD during the baseline (May 2007), 6-week (July 2007), and 1-year (July 2008) followup. The bar indicates the 25% to 75% quartiles for each data. The difference between the baseline value (May 2007) and both of the followup assessments is significant at *P* < 0.0001. The difference between July 2007 and July 2008 is not significant at *P* = 0.07.

 The teachers' follow-up Vanderbilt raw score assessments were completed after 6 weeks in July 2007 and after a year in July 2008. The same 8 teachers who completed the performance impairment assessments also completed these assessments for each of the children in their classes.  Figure 2 shows that the score decreased from the baseline median 13 range 7–21 to median 4 range 1–9 at 6 weeks (*P* < 0.0001 Wilcoxon signed rank test). Though there was further improvement between July 2007 and July 2008, to median 0.5 range 0–14, this was not statistically significant (*P* = 0.07 Wilcoxon signed rank test). 

**Figure 3:**
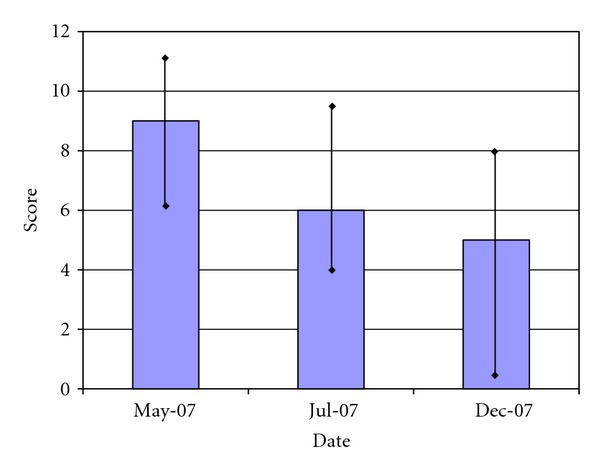
Parent raw Vanderbilt scores for children in Climb-Up Program (*N* = 49). The figure shows the parents' raw Vanderbilt median scores for children in the Climb-Up program at baseline (May 2007), after 6-weeks (July 2007), and 6-month (Dec 2007) followup. The bar indicates the 25% and 75% quartiles for each data. The difference between the baseline value (May 2007) and both of the follow-up assessments is significant at *P* < 0.001. The difference between July 2007 and Dec 2007 is not significant (*P* > 0.05).

 The parents' follow-up assessments were completed in July 2007 for 53 students and December 2007 for only 49 of the students.  Figure 3 shows that the median score decreased from 9, range 4–20 at baseline May 2007 to 6 range 2–18 in July 2007 and 5 range 0–18 in December 2007. Each assessment was significantly better from baseline (*P* < 0.001 Wilcoxon signed rank test). Though there was a small improvement between July 2007 and December 2007, it was not statistically significant (*P* = 0.12 Wilcoxon signed rank test).

According to the parent reports, by December 2007, 4/49 (8.1%) students had ADHD symptoms that remained the same or worsened and 45/49 (91.9%) had improved from baseline. In terms of parent and teacher concordance, agreement in direction of change was seen by both at 6 weeks in 41 children (78%).

 In terms of comorbidities 4/55 (7.2%) had oppositional defiant disorder. Interestingly, ODD comorbidity was present in 8/14 (57%) placed on medications and excluded from this analysis. Neither ODD status nor gender was related to improvements on Climb-Up program. Dyslexia was equally distributed between those on medications and those not and was not related to improvements.

The students lost to followup either moved or the parents were unable to attend for the Vanderbilt parent follow-up day. These students had similar ADHD indices at the beginning with median performance impairment score of 5 range 3–7 and Raw Vanderbilt median score of 9 range 5–10.

## 4. Discussion

 We were able to successfully maintain a peer-mediated multimodal behavioral program throughout the year using local high school volunteers in a large school in a relatively poor urban setting in India. The program called Climb-Up was established over an initial 6-week period [15], efficacy documented, and subsequently managed solely by school resources with no further external support. The core aspects of the program included establishing a one-hour session incorporating yoga and meditation, play therapy, positive reinforcement, and training of local high school volunteers to then maintain the program. 

Performance impairment assessed by teachers showed improvement in academic performances, especially reading, as well as social and peer interactions in most of the children. Both parent evaluations as well as teacher evaluations showed sustained improvements of the behaviors as well, especially in the child's ability to pay attention in class, organizational skills with homework, and decreased impulsive behavior. Though anecdotal, teachers reported unusual events such as children for the first time coming up to them requesting homework assignments! The teacher evaluations confirm that the positive results were maintained from July 2007, 6 weeks after starting the program, through to the following July 2008. 

The fact that the local high school student volunteers sustained this after only a two-week training period is an important finding. Other workers using intensive multimodal approaches to treating ADHD have reported a failure in sustaining initial gains once the intervention has been discontinued [16]. By simplifying the intervention, and using local trained resources such as high school volunteers we have shown the potential of this affordable option in maintaining improvements.

 The children as well as the peer volunteers enjoyed the meditation and yoga and were particularly interested in the play therapy. It was clear that another relationship was developing between the peers and the students beyond these sessions, having a positive impact on the child's self-esteem. Because of the study sample size and the difficulty of evaluation of this social effect, the peer interaction was not directly measured; however it may still have likely been one important factor in the program's success. Further large-scale prospective studies would be needed to properly measure and address this effect.

These results show a remarkable consistency in response to the peer-mediated program. While some of the effects may be credited to initial enthusiasm and attention, the efficacy of the program is evident by the fact that the majority of children reported improved performance in school, which was sustained for the year. Similar results have been shown in larger multimodal studies, albeit with trained psychologists in the USA [14]. One important benefit of embedding the program in the school curriculum is the observed high level of compliance.

While Vanderbilt ADHD questionnaire has been used in India by other researchers, it has yet to be validated in India, especially in illiterate populations who require help. However, it has been validated in many other non-English speaking countries. While this limits the validity of using the tool for diagnosis in our population, longitudinal comparisons for the same child would be less affected. Even if validated, a mediator or translator would have still been required. A clinical assessment by a neurodevelopmental pediatrician was used to make the specific diagnosis of ADHD based on DSM-IV criteria. This remains the gold standard in making a diagnosis and needs to be an integral part of setting these programs up. 

Fourteen children were removed from this study after the initial 6 weeks as they showed an incomplete response, and were placed on medications. Of these 8/14 (57%) had oppositional defiance disorder as a comorbidity, significantly more than 4/55 (7.2%) in the nonmedicated children. Major hurdles had to be overcome to provide Methylphenidate. It is regulated as a controlled substance in India, and none of the local pharmacies agreed to dispense it. Indeed supplies had to be obtained in New Delhi with individual prescriptions through the local pediatrician, and brought to Najibabad, creating significant cost and logistic hurdles. Furthermore, these could only be safely administered at school by designated staff given school board concerns of parental understanding and ability to follow instructions. These children would typically need not only medication but also more skilled behavioral and psychological resources than those that are commonly available in such developing regions. However, after allowing for this incomplete response group (18% of all who initially enrolled), this program offers a simple but cost-effective approach for the majority. 

 The findings of this pilot study, limited because of the small sample size and lack of control group, warrant a larger controlled and ideally multicentered study to confirm efficacy. Additional biomarkers, such as resting heart rate or stress measures that improve with meditation, as well as serial ADHD child administered computerized performance tests that do not rely on parent or teacher evaluations [22] may help further elucidate direct or indirect effects of components of the intervention. Indeed one recent pilot study from Arizona shows preliminary evidence of efficacy of transcendental meditation alone in ADHD in 11 children, reaffirming the potential role of yoga with meditation [23]. Such a study also supports the belief that the multimodal aspects of the Climb-Up program could be adopted for use in the United States or elsewhere internationally. 

 Finally, attention should also be given to the children who failed to improve. Significant long-term local and national healthcare and education reforms would be needed, to train physicians and pharmacists and to employ special education teachers and behavioral and cognitive psychologists. In the interim, one approach that may be considered is to increase the number of sessions per week and to engage the parents or older siblings so they become the teachers and guides for these children at home.

## Appendix

### DSM-IV Criteria for ADHD

(i) Either A or B.

(a) Six or more of the following symptoms of inattention have been present for at least 6 months to a point that is disruptive and inappropriate for developmental level.

Inattention

1. Often does not give close attention to details or makes careless mistakes in schoolwork, work, or other activities.
2. Often has trouble keeping attention on tasks or play activities.
3. Often does not seem to listen when spoken to directly.
4. Often does not follow instructions and fails to finish schoolwork, chores, or duties in the workplace (not due to oppositional behavior or failure to understand instructions).
5. Often has trouble organizing activities.
6. Often avoids, dislikes, or does not want to do things that take a lot of mental effort for a long period of time (such as schoolwork or homework).
7. Often loses things needed for tasks and activities (e.g., toys, school assignments, pencils, books, or tools).
8. Is often easily distracted.
9. Is often forgetful in daily activities.

(b) Six or more of the following symptoms of hyperactivity-impulsivity have been present for at least 6 months to an extent that is disruptive and inappropriate for developmental level.

Hyperactivity

1. Often fidgets with hands or feet or squirms in seat.
2. Often gets up from seat when remaining in seat is expected.
3. Often runs about or climbs when and where it is not appropriate (adolescents or adults may feel very restless).
4. Often has trouble playing or enjoying leisure activities quietly.
5. Is often “on the go” or often acts as if “driven by a motor.”
6. Often talks excessively.

Impulsivity

1. Often blurts out answers before questions have been finished.
2. Often has trouble waiting one's turn.
3. Often interrupts or intrudes on others (e.g., butts into conversations or games).

(ii) Some symptoms that cause impairment were present before age 7 years.

(iii) Some impairment from the symptoms is present in two or more settings (e.g., at school/work and at home).

(iv) There must be clear evidence of significant impairment in social, school, or work functioning.

(v) The symptoms do not happen only during the course of a Pervasive Developmental Disorder, Schizophrenia, or other Psychotic Disorder. The symptoms are not better accounted for by another mental disorder (e.g., Mood Disorder, Anxiety Disorder, Dissociative Disorder, or a Personality Disorder).

Based on These Criteria, Three Types of ADHD Are Identified

1. ADHD, *Combined Type*: if both criteria 1A and 1B are met for the past 6 months
2. ADHD, *Predominantly Inattentive Type*: if criterion 1A is met but criterion 1B is not met for the past six months
3. ADHD, *Predominantly Hyperactive-Impulsive Type*: if criterion 1B is met but criterion 1A is not met for the past six months.
